# Introducing Negatively Charged Residues on the Surface of Fetal Hemoglobin Improves Yields in *Escherichia coli*

**DOI:** 10.3389/fbioe.2021.721794

**Published:** 2021-09-06

**Authors:** Karin Kettisen, Leif Bülow

**Affiliations:** Division of Pure and Applied Biochemistry, Department of Chemistry, Lund University, Lund, Sweden

**Keywords:** protein engineering, fetal hemoglobin (HbF), recombinant production, protein net surface charge, chromatography

## Abstract

Fetal hemoglobin (HbF) has been developed into an important alternative protein for oxygen therapeutics. Such applications require extensive amounts of proteins, which only can be achieved via recombinant means. However, the expression of vertebrate hemoglobins in heterologous hosts is far from trivial. There are several issues that need to be dealt with. These include, among others, the solubility of the globin chains, equimolar expression of the globin chains, and access to high levels of free heme. In this study, we examined the impact of introducing negative charges on the surface of HbF. Three different HbF mutants were examined, carrying four additional negative charges on the *α*-subunit (rHbFα4), two additional negative charges on the γ-subunit (rHbFγ2) or a combination of these (rHbFα4/γ2). The increase in negative surface charge in these HbF mutants required the development of an alternate initial capture step in the downstream purification procedures. For the rHbFα4 mutant, we achieved a significantly enhanced yield of purified HbF with no apparent adverse effects on Hb functionality. However, the presence of non-functional Hb portions in the rHbFγ2 and rHbFα4/γ2 samples reduced the yields significantly for those mutants and indicated an imbalanced expression/association of globin chains. Furthermore, the autoxidation studies indicated that the rHbFγ2 and rHbFα4/γ2 mutants also were less oxidatively stable than rHbFα4 and wt rHbF. The study further verified the need for an improved flask culture protocol by optimizing cultivation parameters to enable yield-improving qualities of surface-located mutations.

## Introduction

Blood transfusion is a routine and lifesaving clinical intervention in many fields, including emergency medicine, advanced surgery, and cancer treatment. However, the shortage of blood donors and the risk of bloodborne pathogens have in many instances hampered regular surgical procedures. Alternatives to blood that can complement and reduce the demands on blood have therefore long been sought after in medicine. Several potential agents have been proposed including hemoglobin-based oxygen carriers (HBOC)s (Alayash 1999) and perfluorocarbons (Castro and Briceno 2010). However, the development of these agents has largely been constrained by safety issues and a lack of efficient large-scale recombinant production systems (Alayash 2014). Safety concerns have been addressed by chemical cross-linking or by PEGylation of Hbs, which have been purified from animal or human RBC (Buehler et al., 2010). These measures prevent the Hb proteins from dissociating into dimers and enhance the circulation time by increasing the molecular size of the native Hb. Additional modifications by protein engineering have been pursued to reduce nitric oxide (NO) binding effects, minimize renal toxicity, and control redox activity (Benitez Cardenas et al., 2019). Such strategies require that Hb proteins can be expressed and produced efficiently in heterologous hosts. However, efforts to enhance recombinant expression techniques in microbial systems have only been partially successful (Zhao et al., 2021).

The expression of vertebrate Hbs in recombinant hosts thus presents several challenges. Due to the tetrameric nature of Hb, combined with the requirement for an adequate supply of the functional prosthetic group heme, it is essential to achieve balanced expression rates of the separate subunits and expedite heme production. Human Hbs are tetramers, made up of four subunits—two *α*-type globins (α, ζ) and two *ß*-type (β, γ, δ, ε) globins, that combine into heterodimers of one *α*-type and one *ß*-type, and then two heterodimers combine into a full tetramer (Schechter 2008). The early recombinant production protocols involved methods where the subunits were recombined *in vitro* after separate cultivation/purification to form the complete tetramer (Nagai and Thøgersen 1987). In the early 1990s, the first successful tandem expressions of the Hb subunits to form complete recombinant human HbA (*α*_2_β_2_) were reported in *Escherichia coli* (Hoffman et al., 1990) and *Saccharomyces cerevisiae* (Wagenbach et al., 1991). However, from the earliest reports on recombinant production of HbA, it was noted that primarily the *α*-subunit was difficult to express (Hoffman et al., 1990). It has been hypothesized that solubility issues could be the reason, related to the inability of the *α*-subunit to form soluble stable structural features in the absence of *ß*-type globins (Oton et al., 1984). Extensive studies of myoglobin have shown that during expression, the stability of the apoglobin is imperative to achieve high yields (Samuel et al., 2015). In the case of Hb, this dependence on the stability of the apo-subunits on yields has not yet been fully confirmed, probably due to the additional subunit association factors complicating the Hb folding/precipitation processes (Olson 2020).

The stability problem of unequal expression of Hb subunits is well-known long before recombinant production in heterologous host cells was developed. For instance, during pathological conditions such as *ß*-thalassemia, where insufficient expression of the *ß*-subunit leads to unbalanced subunit ratios, the aggregation of *α*-subunits causes oxidative damage and apoptosis, which leads to anemia (Cao and Galanello 2010). At normal conditions during erythropoiesis, the molecular chaperone alpha hemoglobin stabilizing protein (AHSP) is present in the maturing red blood cells (RBCs) and binds to the *α*-subunit before association to the *ß*-subunit, thereby relieving the stability issue of individual *α*-subunits and aiding tetramer formation *in vivo* (Gell et al., 2002; Kihm et al., 2002). Co-expression of this chaperone in *E. coli* during recombinant Hb production has been shown to aid the expression of the *α*-subunit (Vasseur-Godbillon et al., 2006). Although it has also been observed that with equimolar ratios of AHSP, *α*-subunit, and *ß*-subunit the AHSP will compete with the binding of *α* to the *ß*-subunit (Faggiano et al., 2011; Varnado et al., 2013). Therefore, to achieve improved expression efficiency of Hb tetramers, the expression ratios of the globin chains must be adapted. In addition, the expression of several proteins in the same host cell needs additional optimization and might become a burden on cellular metabolism.

In the end, the three components required to form functional human Hb molecules are *α*-type globin, *ß*-type globin, and heme. An approach to aid the expression of these proteins in recombinant host systems is to apply protein engineering to increase the solubility and stability of the expressed globins. Inside *E. coli,* the net charge repulsion is imperative to assure dispersal of intracellular components (Mu et al., 2017), and the cytosolic proteome has been reported to exhibit a uniform negative net-charge density of around −1e/11 nm^2^ (Wennerström et al., 2020). In general, regarding the solubility of peptides, hydrophobic patches correlate with reduced solubility, while adding charges to surface-exposed sites can increase solubility. However, positively charged patches are also correlated with increased insolubility, which might be explained by how positively charged areas on proteins correlate to the ability to associate with nucleic acids (Chan et al., 2013). In a bacterial host where there is no nucleus, i.e., no physical separation of the chromosomal DNA and the surrounding cytoplasm, substituting positive charges for negative charges on the protein surface could be a strategy to decrease the chances of DNA association/interference by the recombinant protein, which may be beneficial for the expression system. In the case of Hb, it has been reported that positively charged residues reduce the association of subunits into the full Hb tetramer, while negatively charged residues increase association (Mrabet et al., 1986; Adachi et al., 1998), further supporting the possible benefits of adding negative charges to the surface of the Hb protein for improved recombinant expression.

Examples of mutations that have been shown to increase yield in recombinant fermentation of HbA is the β82 (EF6) Lys→Asp (Hb Providence_asp_ (Moo-Penn et al., 1976)) (Weickert et al., 1999), α15 (A13) Gly→Ala, β16 (A13) Gly→Ala, and β116 (G18) His→Ile (Graves et al., 2008). For an overview of mutational strategies, Olson recently summarized 50 years of research and mutational work on myoglobin and Hb and highlights the importance of polypeptide chain stability at different stages of globin assembly (Olson 2020). However, the impact of net charge density of the surface of Hb regarding yields in recombinant expression has not been the subject of any comprehensive studies to the best of our knowledge. Concerning myoglobin, the most easily expressed myoglobin variants belong to deep-diving mammals (Scott et al., 2000), and a common feature of these stable, highly expressed proteins is an adaptation towards increased net surface charge to maximize myoglobin concentration in the tissue (Mirceta et al., 2013). Hypothetically, employing a similar strategy to the subunits of Hb without disrupting subunit association or basic functionality may lead to a more stable protein capable of better yields in recombinant hosts.

It has previously been demonstrated that human fetal Hb (HbF) offers several advantages that could be useful in the design of HBOCs compared to HbA, notably higher tetrameric strength (Yagami et al., 2002), higher alkaline stability (Perutz 1974), and higher expression levels during recombinant expression in *E. coli* (Ratanasopa et al., 2016). In this work, we decided to explore the effects of exchanging positive surface charges to negatively charged residues on the protein surface of HbF. This study aims to examine a new set of recombinant variants of HbF in terms of expression and basic functionality, including four mutations on the *α*-subunit and two on the γ-subunit. The *α*-subunit mutations involve three positive lysine residues on the surface of the alpha chain that has been substituted for negatively charged glutamate. In addition, an asparagine residue has been mutated to an aspartate. The *α*-subunit mutations are as follows: α11 (A9) Lys→Glu, α56 (E5) Lys→Glu (Hb Shaare Zedek (Abramov et al., 1980)), α78 (EF7) Asn→Asp (Hb J-Singa (Wong et al., 1984)), and α90 (FG2) Lys→Glu (Hb Sudbury). The αK11E mutation was selected from a design perspective in part for its proximity to α12, which is a potential site for adding surface-located negative charge as demonstrated previously (Kettisen et al., 2021). The other mutations have been reported as naturally occurring mutations in the Hb variant database (Giardine et al., 2014). Compared to a previously reported HbF mutant (αA12D/A19D) where two negatively charged residues were placed relatively closely on the A helix (Kettisen et al., 2021), the mutation sites presented here were selected also to provide a more dispersed spread of negative charges on the surface of the *α*-subunit. In addition to introducing negative charges on the *α*-subunit, the γ-subunit was modified similarly. The γ-subunit mutant prepared carries two mutations: γ61 (E5) Lys→Glu, previously described as HbF Jamaica (Ahern et al., 1970), and γ82 (EF6) Lys→Asp. In total, three HbF mutants are evaluated in this work and will hereafter be denoted as rHbFα4, rHbFγ2, and rHbFα4/γ2, respectively.

Apart from evaluating the expression levels of these Hb mutants, we also examined the chromatographic behaviors of the proteins. Hb is normally purified from a biological matrix, RBC, or recombinant hosts, by ion exchange resins (Reeder et al., 2008; Sun and Palmer 2008; Natarajan et al., 2011; Ratanasopa et al., 2016). However, since the surface charges are modified in our mutants, the regularly utilized purification protocols for our HbF mutant proteins (Kettisen et al., 2018) are no longer efficiently applicable. In this study, we developed a new purification procedure for the more negatively charged HbF mutants based on an anion exchange multimodal chromatography resin. It is thereby possible to pinpoint the influence of surface charges on a target protein and thereby develop highly efficient methods for protein purification (Kallberg et al., 2012).

## Materials and Methods

**FIGURE 1:**
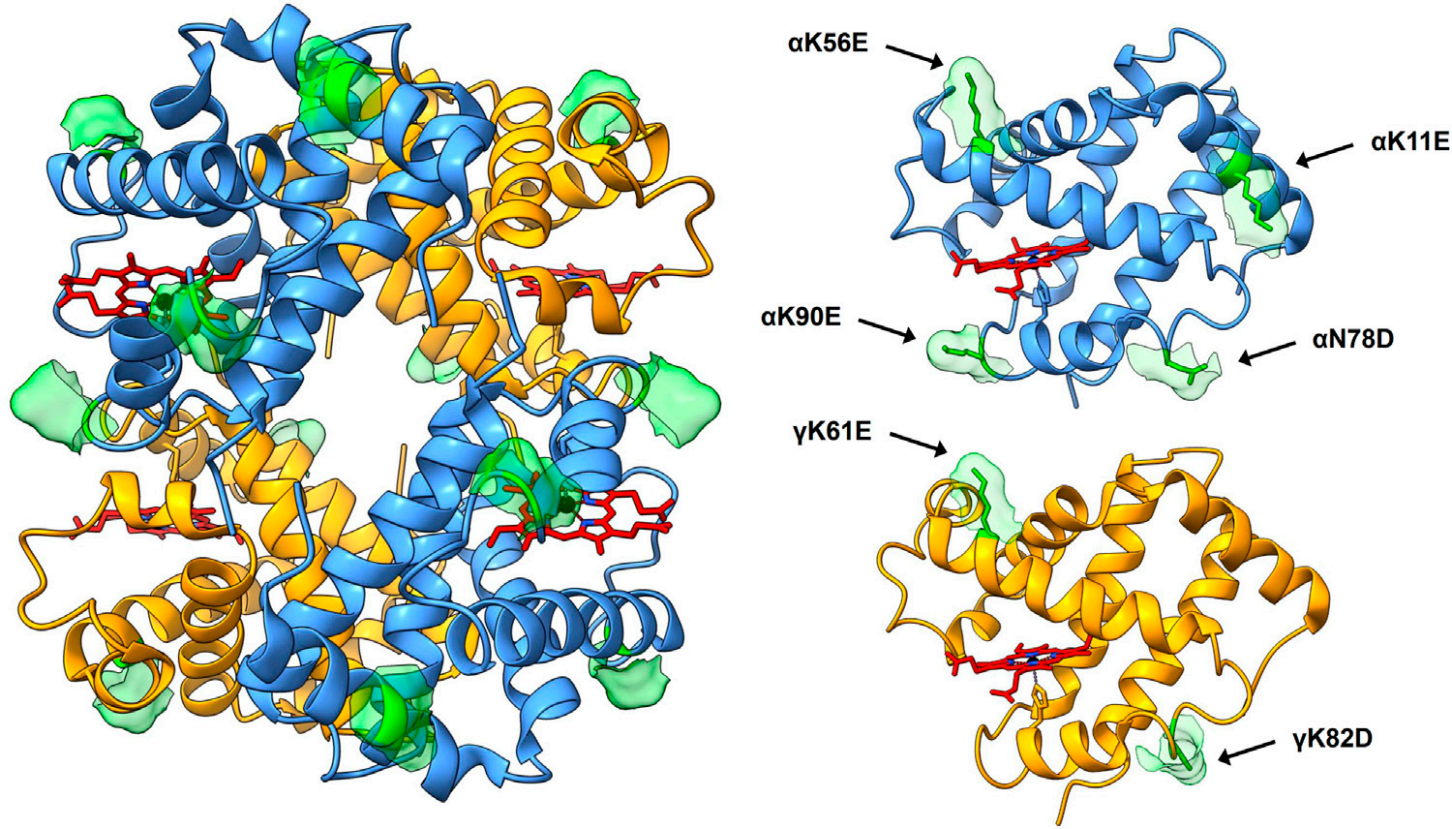
3D structure of HbF (PDB code 1FDH (Frier and Perutz 1977)). The full tetramer structure is shown to the left, with the peptide backbone depicted as ribbons. *α*-subunits are colored blue, *γ*-subunits yellow, and heme groups are shown as stick figures in red with blue nitrogen and black iron atoms. The mutation sites of the HbF mutants created in this work are shown in green, accompanied by the corresponding solvent-accessible surface patch to highlight the sites. To the right, the *α*- and *γ*-subunit are shown for clarification of the positioning of the mutation sites. The figure was created in the UCSF Chimera molecular visualization program (Pettersen et al., 2004; Goddard et al., 2018).

The Hb variant database (Giardine et al., 2014) was searched for natural mutations resulting in substitutions from positively to negatively charged residues. Mutation sites were chosen according to reported retained normal functionality/stability and/or placements on the tetrameric structure of HbF assessed by published crystal structures of wild-type (wt) HbF (Frier and Perutz 1977; Soman and Olson 2013). The genes for the mutated *α*- and γ-subunits were codon-optimized for *E. coli* B strain and obtained from Integrated DNA Technologies (IDT, Germany). The DNA fragments were cloned into a previously described HbF-pETDuet-1 vector for tandem expression of the globin genes (Ratanasopa et al., 2016) to form three HbF variants; 1) modified *α*-subunit carrying four additional negative charges together with wt γ-subunit (rHbFα4), 2) modified γ-subunit carrying two additional negative charges together with wt *α*-subunit (rHbFγ2), and 3) both modified subunits together (rHbFα4/γ2). The modified alpha chain harbored the four substitutions αK11E, αK56E, αN78D, αK90E; and the gamma chain had two mutations γK61E, γK82D (Figure 1).

### Cultivation Optimization

The modified pETDuet-1 vectors were transformed into *E. coli* BL21 (DE3) cells and two cultivation screenings were performed using the rHbFα4/γ2 variant harboring both mutated subunits to screen for improved expression conditions. Five cultivation parameters were screened: incubator temperature, gas exchange by varying rotation speed of the cultures, optical density at the time of induction, heme precursor concentration, and inducer concentration. The starter cultures were grown overnight in LB (Luria-Bertani) medium supplemented with 100 μg/ml carbenicillin at 37°C. The main culture medium was TB (Terrific Broth), also supplemented with carbenicillin, as well as δ-aminolevulinic acid (ALA) as heme precursor and isopropyl-β-D-thiogalactoside (IPTG) for inducing expression. The cultures were grown in 150 ml TB in 500 ml Erlenmeyer flasks with indented baffles and sealed tightly with cork and parafilm around the edges. In the first screening round, all five parameters were varied in a fractional factorial factor III design setup, while the second round had fixed induction OD_600_ and temperature, while alternating rotation speed, ALA, and IPTG concentration in a full factorial setup. The cells were harvested by centrifugation 16.5 h after inducing protein expression and then washed twice in 50 mM Tris-HCl buffer pH 8.5. The cells were ruptured by sonication, and then centrifuged and filtered to remove cell debris. The concentration of Hb in the clarified crude extracts was estimated by assessing the Hb-CO spectrum in a Cary 60 UV-Vis spectrophotometer (Agilent Technologies), using the extinction coefficient of the peak at 419 nm for Hb-CO (Antonini and Brunori 1971). The height of the 419 nm peak was adjusted with a three-point drop calculation to adjust for the inclined background of the crude protein extract.

### Liquid Chromatography Purification

The rHbFα4/γ2 variant was used to screen for an alternative purification resin for the first step in the downstream processing of the mutants, as well as for optimizing the chromatographic conditions for the mutant HbF variants, using an ÄKTA Avant 25 chromatography system (GE Healthcare). HiTrap columns DEAE FF, Q FF, and Chelating FF charged with Ni^2+^ (GE Healthcare), and BabyBio DEAE, Q, and TREN (Bio-Works, Sweden) were examined as candidate resins. TREN, a multimodal ion exchange resin composed of rigid agarose beads functionalized with Tris (2-aminoethyl)amine ligands (WorkBeads™ 40 TREN, Bio-Works, Sweden) was chosen as the new resin material for the initial capture step at the end of the trial purification runs. A larger-scale setup with 75 ml TREN material was packed in a HiScale 26/20 column (GE Healthcare) for purification of greater sample volumes. The mutated HbF variants were loaded onto the TREN column equilibrated with 20 mM Tris-HCl pH 8.5. A short salt gradient to 200 mM NaCl in Tris-HCl buffer was used as an extra washing step and then followed by a buffer change to 50 mM MES buffer pH 5.6. Finally, a linear salt gradient to 500 mM NaCl was used to elute the mutant HbF proteins. The samples were then concentrated on Vivaspin columns (30,000 MWCO, Sartorius), and buffer-exchanged to 20 mM Tris-HCl pH 7.4 on PD-10 desalting columns (Sephadex G-25, GE Healthcare). The second purification step resin was practically unchanged from previous protocols (Q HP, GE Healthcare) (Ratanasopa et al., 2016; Kettisen et al., 2018). Elution was performed with the same buffer supplemented with NaCl in a linear gradient. Initial quality control of the Hb fractions was performed by adding 25x molar excess of K_3_ [Fe(CN)_6_] (Sigma-Aldrich) to the eluted fractions and the spectral change was followed every minute for 20 min. The Hb sample purity was assessed densitometrically after the two chromatography steps by sodium dodecyl sulfate polyacrylamide gel electrophoresis (SDS-PAGE). The purified samples were concentrated to 2 mM (heme-based) and flash-frozen in liquid nitrogen and stored at −80°C before further characterization.

### Hb Characterization

The functionality of the mutant HbF was assessed several-fold by spectrophotometric analysis of the anticipated ligand states, autoxidation, and isoelectric point determination, respectively. The different spectra were produced according to previously described methods (Kettisen et al., 2021). Briefly, the HbF mutants were examined on a Cary 60 UV-Vis spectrophotometer (Agilent Technologies) in different ligand-bound and oxidation states of hemoglobin: Hb-CO, Hb-O_2_, deoxy Hb (Fe^2+^), and ferric Hb (Fe^3+^). Hb-CO spectrum was achieved by bubbling the Hb samples with CO gas. The Hb-O_2_ spectrum was obtained by placing the Hb under a bright light source and continuously exposing the samples to a steady stream of pure O_2_ gas until no Hb-CO remained. Ferrous deoxy Hb (Fe^2+^) spectrum was recorded after adding excess sodium dithionite (Sigma-Aldrich) to the Hb-O_2_ for complete O_2_ removal. The ferric Hb (Fe^3+^) spectrum was obtained by adding potassium ferricyanide (K_3_ [Fe(CN)_6_], Sigma-Aldrich), incubating on ice until the oxidation reaction was complete, and then removing the excess oxidant with buffer exchange on a Sephadex G-25 desalting column (GE Healthcare). The obtained spectra were compared to reported data (Antonini and Brunori 1971; Meng and Alayash 2017). Autoxidation was performed by converting the protein samples to the O_2_-bound form with pure O_2_-gas under strong light (n = 3), followed by buffer exchange on PD-10 desalting columns (Sephadex G-25, GE Healthcare) to 100 mM phosphate buffer pH 7.4. Subsequently, the spontaneous reaction of 10 µM Hb at 20°C was followed by recording the spectra every 30 min for 24 h. The final ferric spectrum was achieved by K_3_ [Fe(CN)_6_-induced oxidation to obtain the reaction end point. The acquired spectral series were processed with component analysis in the 450–700 nm range (Singh et al., 2020), and the ferrous decay time course was fitted to a single exponential equation with least square fitting in Microsoft Excel program with the Solver add-in. Statistical differences were determined by independent samples *t*-test (*p* < 0.5). The isoelectric focusing was performed in a Novex^®^ pH 3–10 IEF gel (Invitrogen) with IEF Standards (Bio-Rad), as well as under denaturing conditions in 8 M urea, using Immobiline DryStrip pH 3–10 (7 cm), run in an IPGphor Isoelectric Focusing System (Pharmacia).

## Results

Three new variants of HbF were obtained by cloning the *α*- and γ-subunit gene fragments carrying the chosen mutations into the pETDuet-1 vector and transforming the plasmids into *E. coli* BL21 (DE3). The correct insertion of the mutated gene sequences was confirmed by Sanger sequencing (GATC, Eurofins, Germany).

### Cultivation Optimization

An initial cultivation trial using the previously used protocol for HbF expression (Ratanasopa et al., 2016) indicated that protein production for the three mutants could be further optimized. In general, low and varying yields during recombinant Hb production in shake flasks have previously been observed (Kettisen et al., 2018; Simons et al., 2018), and an updated shake flask protocol was hypothesized to improve yields and efficiency of obtaining mutants of HbF. For this study, we chose to carry out the optimization using the rHbFα4/γ2 mutant since this variant was the most different from wt rHbF in terms of the net surface charge. Two screening rounds of alternating five cultivation parameters were performed to improve the protocol. Table 1 presents the experimental parameters and results.

**TABLE 1:** DoE screening of flask culture parameters for Hb expression.

1st screening									
*Parameters*						*Results*			
exp no	Induc. OD	Temp [°C]	rpm	ALA [mM]	IPTG [mM]	Final OD	Cell weight [g]	Hb [µM]	yield_new_/yield_old_
1	0.1	37	150	0.1	0.05	14.9	2.1	21.9	0.7
2	0.1	23	50	0.1	0.2	1.5	0.4	8.8	0.2
3	4	37	50	0.1	0.05	4.3	0.9	19.8	0.5
4	4	23	150	0.1	0.2	10.4	3.6	9.5	0.5
5	0.1	23	150	0.5	0.05	6.2	1.7	56.7	1.4
6	0.1	37	50	0.5	0.2	1.8	0.4	2.9	0.1
7	4	23	50	0.5	0.05	3.9	1.0	18.4	0.5
8	4	37	150	0.5	0.2	9.2	1.6	129.6	3.0
9*	2	30	100	0.3	0.125	5.2	1.3	61.1	1.3
10*	2	30	100	0.3	0.125	5.0	1.3	59.5	
11*	2	30	100	0.3	0.125	5.5	1.3	45.6	
***2*nd*screening*** (*induction OD*_*600*_ *= 4, and incubation temperature = 35 °C*)									
*Parameters*					*Results*				
exp no	rpm	ALA [mM]	IPTG [mM]		final OD	cell weight [g]	Hb [µM]	yield_new_/yield_old_	
1	100	0.4	0.1		5.0	1.4	58.0	1.3	
2	180	0.4	0.5		7.5	1.9	88.7	2.6	
3	180	0.4	0.1		6.9	2.0	171.2	5.2	
4	100	0.4	0.5		6.3	1.9	84.0	2.4	
5	180	1	0.1		7.4	1.9	185.1	5.1	
6	100	1	0.5		5.2	1.3	95.5	2.3	
7	100	1	0.1		5.6	1.7	140.6	3.8	
8	180	1	0.5		7.2	1.8	186.3	5.0	
9*	140	0.7	0.3		7.0	1.9	222.4		
10*	140	0.7	0.3		8.4	1.9	227.4	5.8	
11*	140	0.7	0.3		6.0	1.7	182.1		

*center point replicates. Five parameters were screened: optical density at the time of induction, OD_600_ 0.1–4; temperature during expression phase, 23–37°C; agitation by rotation speed of the shake flasks, 50–180 rpm; ALA (heme precursor) concentration, 0.1–1 mM; and IPTG (inducer) concentration, 0.05–0.5 mM.

The first round showed that the highest Hb concentration was seen with incubation temperature at 37°C, induction at OD_600_ 4.0, 150 rpm shaking speed, 0.5 mM ALA, and 0.2 mM IPTG. A higher concentration of ALA appeared especially favorable in combination with higher shaking speed. Lower yields were typically observed at low shaking speed, especially in combination with induction at low OD.

**FIGURE 2:**
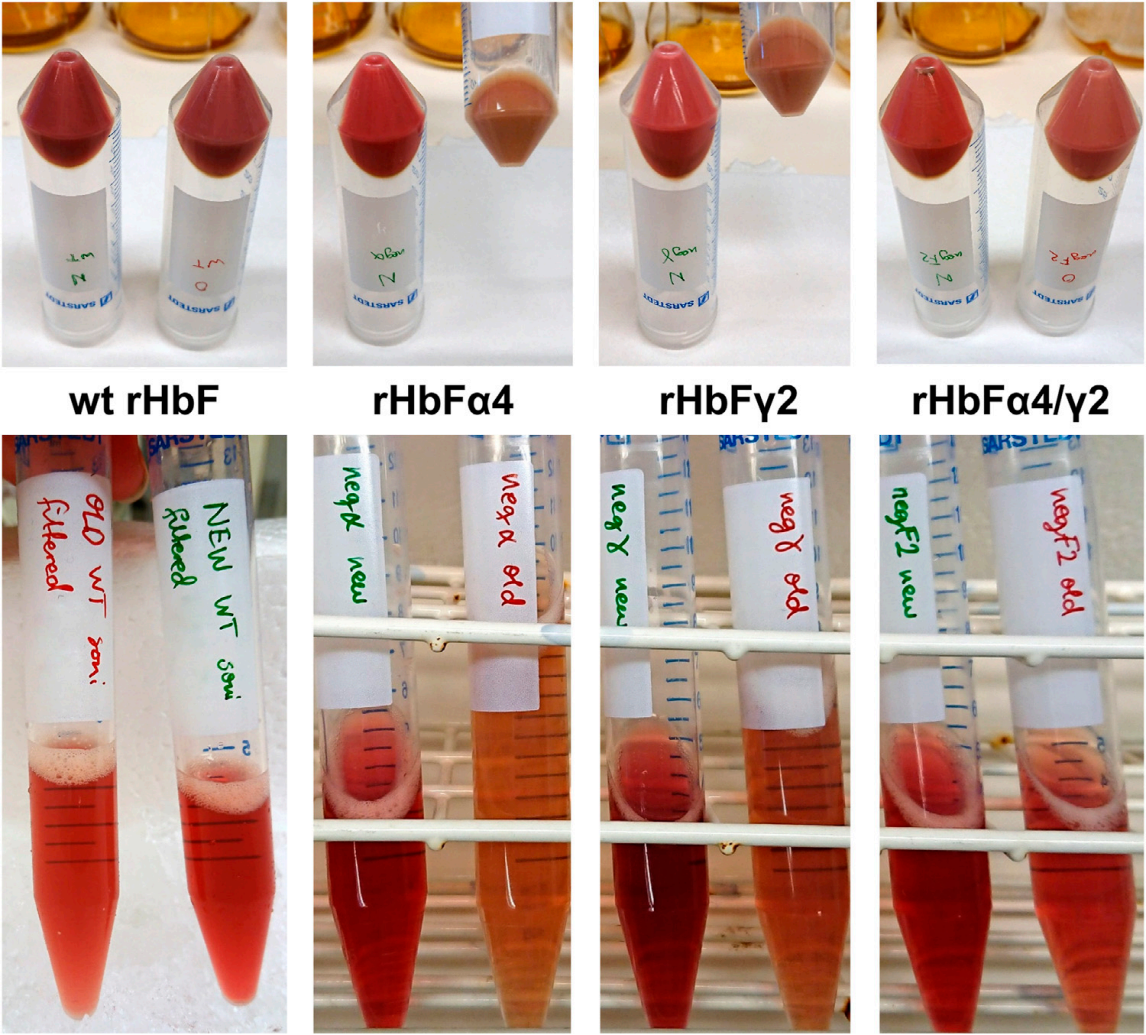
Photos of cell pellets and crude extracts of the HbF variants wt rHbF, rHbFα4, rHbFγ2, and rHbFα4/γ2, cultivated in parallel either with a previously established shake flask protocol (OD_600_ 0.1 at induction, 30°C, 150 rpm, 0.3 mM ALA, and 0.1 mM IPTG) or the improved protocol (OD_600_ 4 at induction, 35°C, 140 rpm, 0.7 mM ALA, and 0.3 mM IPTG). The cultures grown with the improved protocol had higher Hb-CO concentrations in the crude extracts, as also can be seen by the more intense red color in those samples.

In the second screening cycle, the induction OD and temperature were kept at the same conditions, while varying shaking speed, ALA and IPTG. Within the ranges examined, IPTG concentration did not seem to be a significant parameter. Shaking speed, however, seemed to matter, contributing to agitation and increased gas exchange, resulting in increased yields at higher rpm. Compared to the average Hb concentration in *E. coli* crude extracts achieved with the previous protocol for HbF expression, the best conditions of the first screening round showed a three-fold higher Hb yield in the crude extract, while the second screening round increased yield even more with the best conditions. To confirm the result, we compared the previously used protocol with the center point conditions from the second screening round on all HbF variants, wt rHbF, rHbFα4, rHbFγ2, and rHbFα4/γ2. All proteins were expressed better with the new protocol, as can be viewed in Figure 2 by the red color intensity in the pelleted cells and sonicated crude extracts. The calculated concentration of Hb-CO in the crude extracts showed a ∼60% increase for the wt rHbF and up to 4–5 times more Hb in the mutant samples with the new protocol.

### Liquid Chromatography Purification

**FIGURE 3:**
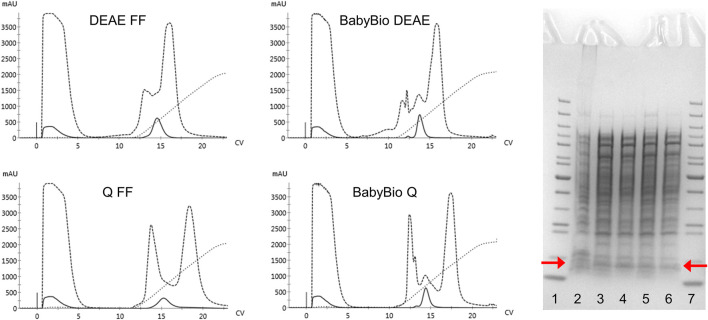
Four chromatograms display the purification procedures on rHbFα4/γ2 from *E. coli* crude extracts in 1 ml columns of different anion exchange resins (HiTrap DEAE FF and Q FF (GE Healthcare) and BabyBio DEAE and Q (Bio-Works)). Solid lines indicate the 419 nm UV trace, dashed lines indicate 280 nm, and dotted lines show the increase in conductivity during elution. The columns were equilibrated with 20 mM Tris-HCl buffer pH 8.0 and elution was performed with a NaCl gradient up to 1 M. The SDS-PAGE gel to the right of the chromatograms shows the protein crude extract and the collected Hb-containing fractions after the chromatographic runs (red arrows indicate the location of the Hb monomer band). The wells contain: 1) PageRuler™ Unstained Protein Ladder (ThermoFisher), 2) rHbFα4/γ2 crude extract, 3) BabyBio DEAE elution, 4) BabyBio Q elution, 5) DEAE FF elution, 6) Q FF elution, 7) PageRuler™ Unstained Protein Ladder (ThermoFisher).

The regular purification protocols for Hb from RBC or recombinant cell extracts are largely dependent on different ion-exchange chromatography steps. The isolation of mutants carrying modified surface charges, therefore, needs to be modified and different chromatographic matrices examined in terms of binding and retention behaviors. The established HbF purification protocol utilized CaptoS (GE Healthcare), which is a strong cation exchanger, as the initial capture step resin (Kettisen et al., 2018). When applying the rHbFα4/γ2 mutant from a crude extract onto the CaptoS resin the mutant HbF did not bind and eluted with the bulk of the contaminating *E. coli* proteins (chromatogram is not shown). This verified that the charge patterns on the protein surface had been modified. An attractive option for Hb purification is based on immobilized metal ion chromatography (IMAC) since HbF carries several surface-located histidine residues. However, a chelating resin charged with Ni^2+^ did not provide sufficient binding capacity to retain the rHbFα4/γ2 mutant and purification yields were negligible (chromatogram is not shown). Next, we examined anion exchange with DEAE and Q columns, where the rHbFα4/γ2 successfully bound to the resin in the capture step and remained attached to the column during the washing step. Elution was achieved with a NaCl gradient ranging up to 1 M. Still, many other host cell proteins contaminated the eluted Hb fractions. The highest purity was achieved with the Q FF resin, but purity was estimated at only ∼9–15% Hb for all resins through analysis on an SDS-PAGE gel. Figure 3 shows the chromatograms from the anion exchange columns, together with the SDS-PAGE gel of the eluted Hb-containing samples.

**FIGURE 4:**
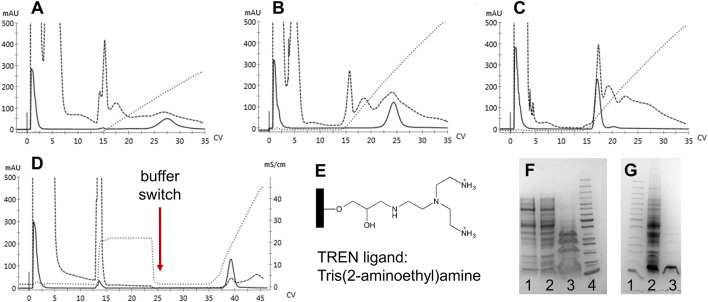
Chromatograms of 1 ml BabyBio TREN chromatographic runs are shown in A-D. Dashed lines indicate the 280 nm UV trace, solid lines indicate 419 nm, and dotted lines show the increase in conductivity during elution. rHbFα4/γ2 crude extracts were loaded onto to column equilibrated with A) 20 mM Tris-HCl pH 8.5, B) 20 mM Tris-HCl pH 7.4, and C) 50 mM MES pH 5.6, and eluted with NaCl gradients. In D) the combined method is shown, where binding of rHbFα4/γ2 is done with 20 mM Tris-HCl pH 8.5, followed by a NaCl wash before the buffer switch to 50 mM MES pH 5.6 and the subsequent elution with NaCl gradient. Panel E) shows the chemical structure of the TREN ligand attached to the agarose beads of the resin. SDS-PAGE gels are shown in F and G. F) 1: TREN pH 8.5 elution, 2: TREN pH 7.4 elution, 3: TREN pH 5.6 elution, and 4: PageRuler™ Unstained Protein Ladder (ThermoFisher). G) 1: PageRuler™ Unstained Protein Ladder (ThermoFisher), 2: rHbFα4/γ2 crude extract, and 3: combined TREN method elution (chromatogram D elution).

Since significant amounts of contaminants remained after elution, the anion exchange resins were deemed unsuitable as replacements for the capture step to compensate for the role of CaptoS. Therefore, we examined a multimodal anion exchange resin, TREN (Bio-Works). The ligand is depicted in Figure 4E. Three different binding buffers with NaCl gradient elutions were evaluated, and with an optimized purification protocol incorporating a buffer exchange and pH shift, a >90% purity of the mutant HbF in the eluted fractions was achieved (Figure 4).

**FIGURE 5:**
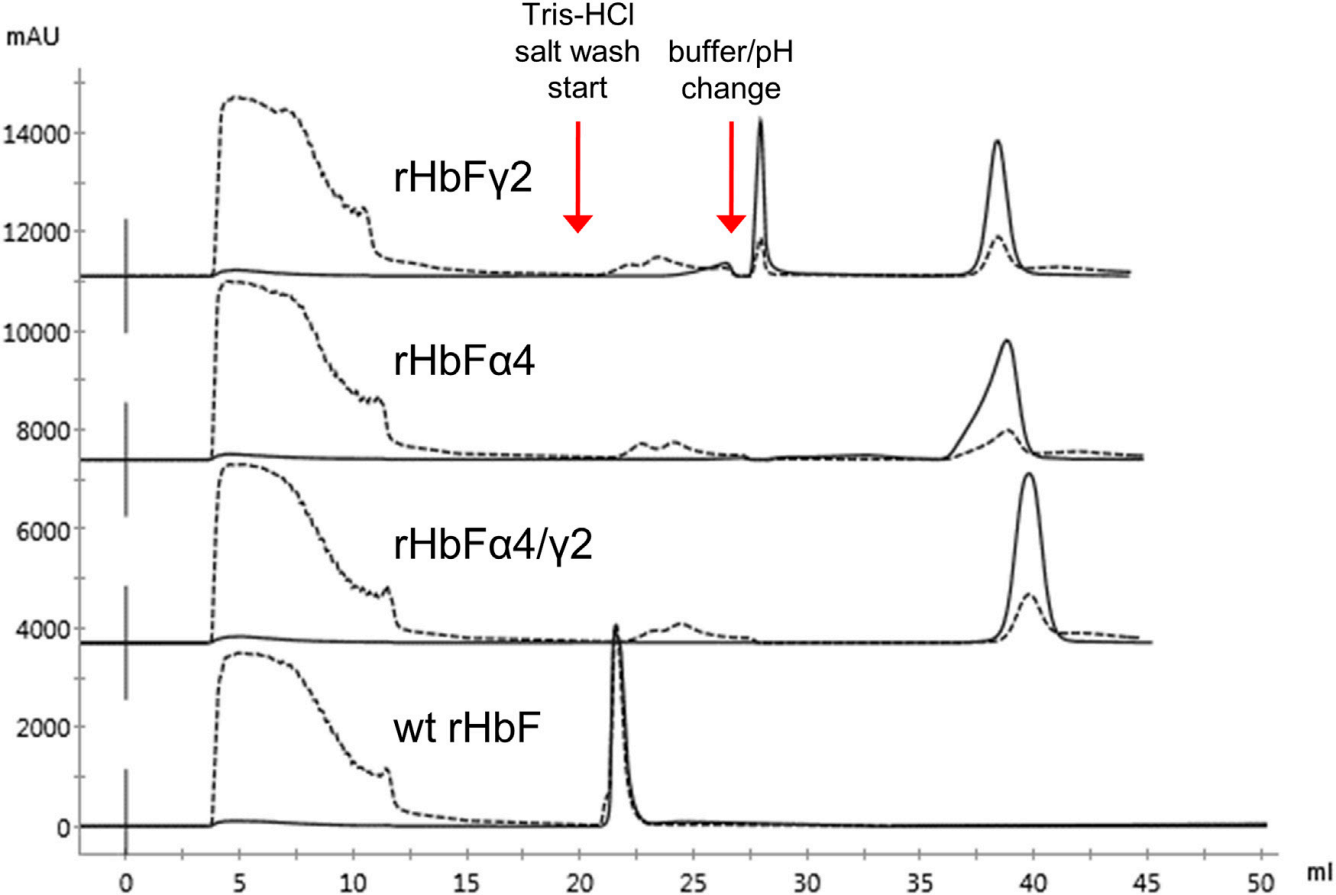
Chromatograms of TREN purification using the pH switch method described previously of wt rHbF, rHbFα4, rHbFγ2, and rHbFα4/γ2. The rHbFγ2 mutant displayed two strong Hb peaks, one during the pH switch step and the other during the final NaCl elution step, while rHbFα4 and rHbFα4/γ2 eluted as one peak fraction at the final elution. The wild-type protein eluted with several contaminating proteins during the first NaCl wash. Solid lines indicate the 419 nm UV trace and the dashed lines indicate 280 nm.

The crude extracts of the wt rHbF and the two other mutants were also applied to the TREN column. The wt rHbF sample eluted in the second washing step from the resin together with host cell contaminants (Figure 5). To achieve sufficient purity of wt rHbF for the subsequent experiments, we continued to use the CaptoS resin for the first purification step of wt rHbF. The rHbFα4 and rHbFγ2 mutants bound readily to the TREN column, but we noted some differences in retention behavior, which were most pronounced for the rHbFγ2 mutant. The TREN purification of the rHbFγ2 mutant yielded two distinct Hb peaks, one during the buffer change step to the MES buffer, and a second peak during the elution step. Examination of the two peak fractions with K_3_ [Fe(CN)_6_] showed that the first peak appeared to contain the functional Hb fraction, while the second fraction quickly formed a hemichrome spectrum when challenged with the oxidant, suggesting a non-functional Hb species. This indicated that the TREN purification procedure was able to effectively distinguish between different Hb fractions within the same crude extract. In contrast, the rHbFα4 mutant only showed a single peak during the TREN chromatography step, which also was deemed as functional Hb with the abovementioned quality control. However, visually, slow migration of the bound red fraction on the TREN column was observed, both at the second washing step and during the MES buffer exchange step, which contributed to a slightly deformed shape of the final elution peak (Figure 5). During repeated cultivation and purification of the rHbFα4/γ2 mutant, we observed from time to time a similar peak as seen with the rHbFγ2 mutant, but for this variant, the first peak was smaller, and deemed non-functional, while the second peak eluted in the elution step showed the functional behavior as confirmed by the K_3_ [Fe(CN)_6_]-method. Combined with the second step column packed with Q HP, the purification of the HbF variants reached a >95% purity of the monomer bands.

We examined the option to exclude the extra washing step and found that an acceptable purification can be performed without the extra Tris buffer salt wash step. This was useful for the purification of rHbFα4 to avoid unwanted migration on the column during the washing steps. However, for the rHbFγ2 and rHbFα4/γ2 samples, it was important to pay extra attention and effort to the elution conditions with the Q HP column to separate the non-functional fractions when the extra wash step on the TREN column was opted out.

With the risk of unwanted Hb variants forming during production, the purification columns must be loaded with an appropriate amount of protein to allow for the detection and separation of diverse Hb peaks. The successful separation of functional and non-functional Hb fractions on Q HP resin has been reported previously (Ratanasopa et al., 2016). Through mass spectrometric examination of the non-functional fraction peak during HbA separation on Q HP, it was determined that this hemichrome-forming species contained only *ß*-subunits (unpublished results). Compared to the heterotetramers of human Hbs, both the *ß*_4_-homotetramer and the γ_4_-homotetramer are more prone to assume the hemichrome form in the presence of excess ferricyanide (Rachmilewitz et al., 1971; Rachmilewitz and Harari 1972). The non-functional fractions seen in two of the three HbF variants in this work could therefore be a homotetramer species consisting of four γ-subunits, as this configuration is also able to form soluble tetramers, a variant called Hb Bart’s (Hunt and Lehmann 1959).

**FIGURE 6:**
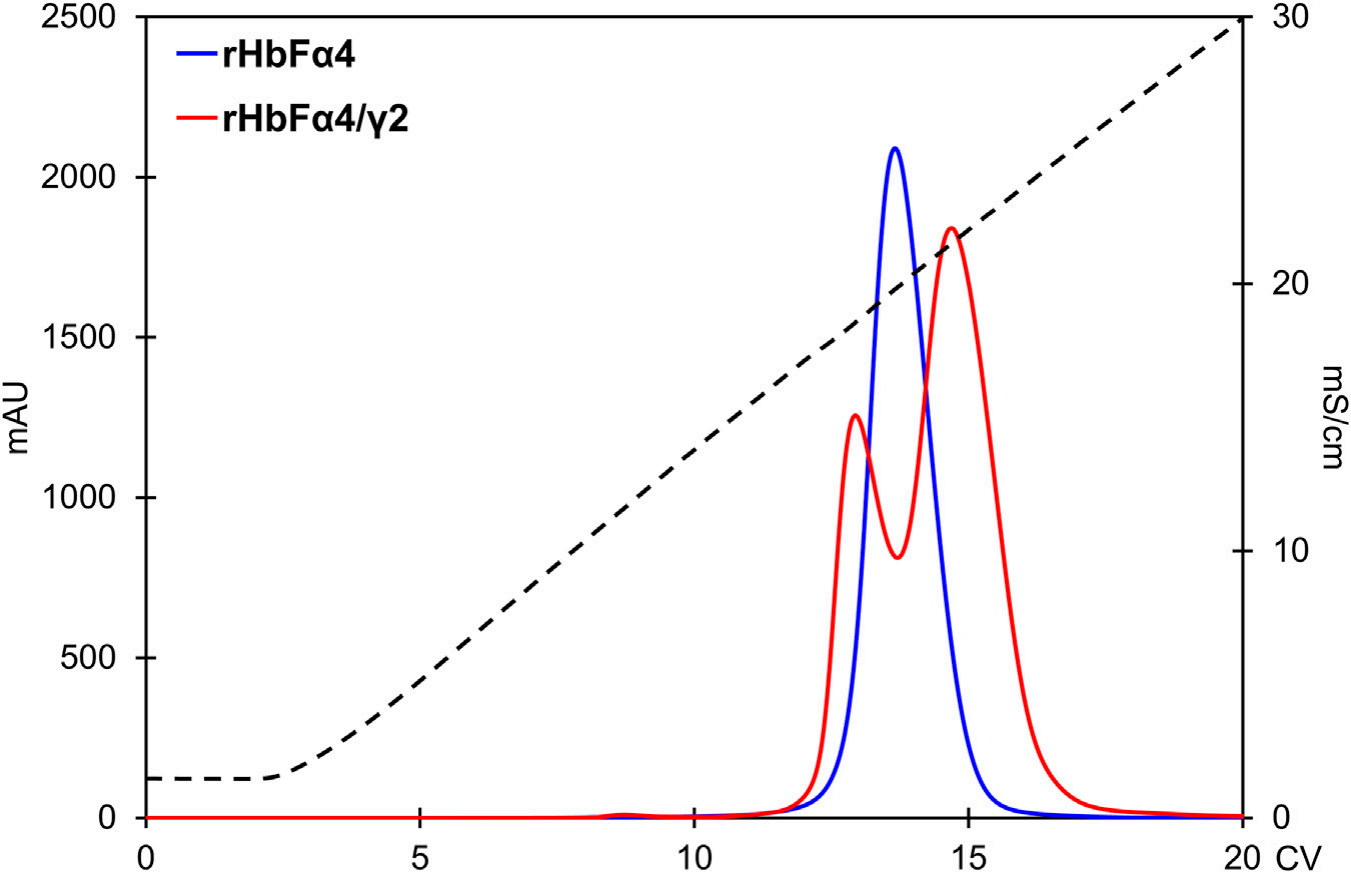
Comparison of the rHbFα4 (blue) and rHbFα4/γ2 (red) elutions during Q HP separation (in this example no extra wash step was used in the capture column TREN prior to the Q HP). The rHbFα4 sample elutes as one homogenous peak, while rHbFα4/γ2 display two peaks. In the rHbFα4/γ2 fractions, the first peak showed hemichrome spectrum when challenged with K_3_ [Fe(CN)_6_] and the second peak displayed the expected ferric spectrum indicating functional behavior. Thus, the rHbFα4/γ2 mutant yield of functional Hb was much less than anticipated from the crude extract calculation, as the sample contained diverse Hb fractions.

As expected from increasing the negative charge on the HbF protein, the NaCl concentrations needed for elution from the Q HP column increased accordingly. The wt rHbF is typically eluted with a Hb peak maximum at around 6 mS/cm (conductivity) and thus does not require high concentrations of NaCl to abolish the binding. In contrast, the three mutants were eluted at 16 mS/cm (rHbFγ2), 19 mS/cm (rHbFα4), and 22 mS/cm (rHbFα4/γ2), showing that significantly higher salt concentrations were needed to efficiently elute these variants from the anion exchange resin. The non-functional fractions of rHbFγ2 and rHbFα4/γ2 had peak maxima at ∼18 mS/cm. Compared to the previous protocol, where the elution step from Q HP involved a gradient from Tris buffer to sodium phosphate buffer supplemented with NaCl, the pure salt gradient in Tris buffer employed in this work gave a linear conductivity slope which enabled a more accurate determination of peak elution conductivity (Figure 6).

### Biophysical Characterization of the Hb Variants

**FIGURE 7:**
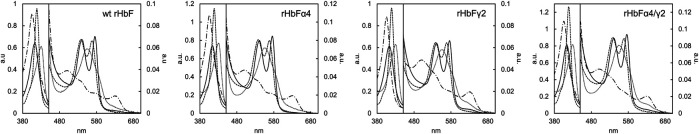
Absorbance spectra of the HbF variants wt rHbF, rHbFα4, rHbFγ2, and rHbFα4/γ2. The solid line represents Hb-O_2_, the dotted line represents deoxy Hb, the dashed line represents Hb-CO, and the dashed-and-dotted line represents oxidized ferric Hb (Fe^3+^).

Hb proteins are normally characterized by spectroscopic examination. All the different spectra of the HbF variants could be obtained with the standard protocols using pure O_2_, CO, sodium dithionite, and K_3_ [Fe(CN)_6_]. Figure 7 shows the spectra of the HbF mutants. None of the mutants appeared to differ significantly from the wt rHbF spectra, as the expected peaks for all forms were obtained (Antonini and Brunori 1971; Meng and Alayash 2017).

The autoxidation study showed that no significant difference was seen between wt rHbF (0.0056 ± 0.0003 h^−1^) and rHbFα4 (0.0050 ± 0.0005 h^−1^), but the two mutants rHbFγ2 (0.0096 ± 0.0002 h^−1^) and rHbFα4/γ2 (0.0085 ± 0.0003 h^−1^) exhibited increased rates of spontaneous autoxidation compared to wt rHbF, indicating less oxidatively stable Hb.

To quantify the impact of the mutation on the overall net charge of the HbF protein, isoelectric focusing was used to assess the effects of the introduced surface charges. The obtained pI values for the mutants were 6.5 for rHbFγ2, 5.8 for rHbFα4, and 5.0 for rHbFα4/γ2, compared to 7.1 for the wt rHbF. The IEF focusing under denaturing conditions with 8 M urea on Immobiline Drystrips showed two bands for the Hb samples. In the wt rHbF sample, the two bands were located relatively close at ∼7.6–7.7, which was a bit unexpected since the estimated pI of the γ-subunit is lower than for the *α*-subunit. One could speculate if this could be due to some interaction between subunits which caused a tandem migration despite the denaturing conditions. The bands of the mutants were more prominently separated. The single subunit mutants showed bands at ∼7.7, together with 7.0 and 6.7 for rHbFα4 and rHbFγ2, respectively, while the rHbFα4/γ2 mutant had bands at 7.0 and 6.7. We also loaded the non-functional fraction of rHbFγ2 on the Drystrips, and this sample only showed one band at 6.7. This further indicated that the non-target Hb species was a homotetramer of γ-subunits. The isoelectric focusing study shows that the mutations contribute to lowering the pI for the HbF mutants while using denaturing conditions during IEF is useful for confirming the presence of both subunits in a sample.

## Discussion

As initially hypothesized, the major finding of this study is that replacing positively charged surface-exposed residues for negatively charged residues appears to affect the expression yields of HbF. For the rHbFα4 mutant, we achieved a significantly enhanced yield of HbF with no apparent adverse effects on Hb functionality. However, the presence of non-functional Hb portions in the rHbFγ2 and rHbFα4/γ2 cultures reduced the yield significantly for those mutants and implied non-balanced association/expression of parts of these samples. Furthermore, the autoxidation studies indicated that the rHbFγ2 and rHbFα4/γ2 mutants also were less oxidatively stable than rHbFα4 and wt rHbF. The study identified the need for an improved flask culture protocol to moderate the limiting cultivation parameters on recombinant Hb yield in *E. coli* to enable assessment of the yield-improving qualities of surface-located mutations. In summary, the mutants of HbF with significantly increased negative net surface charge could be expressed and purified successfully. Even though the basic ligand binding properties were intact, the mutations on the γ-subunit appeared to enhance a bias towards forming non-functional Hb fractions during expression and increased autoxidation rates.

Increased HbF yield in flask cultures with increased temperature, agitation, and precursor concentration. Recombinant production yields in flask cultures in this work showed the potential of adding negative charges on the surface of HbF. Based on the determination of Hb concentrations in the crude extracts, product concentrations could be increased significantly. Over the years, cultivations of recombinant Hb have shown relatively low yields compared to other heterologously produced proteins (Olson 2020). Partly, this can be ascribed to the need for balanced dual expression of the Hb subunits, together with the requirement for an adequate supply of heme. Through numerous expression studies with the previously established protocols for recombinant HbF production (Ratanasopa 2015; Kettisen et al., 2018), we suspected that the cultivation parameters for flask culture expression could be optimized to improve yields. By employing only two screening rounds of five parameters, we improved the crude extract Hb concentration of the rHbFα4/γ2 mutant almost six-fold compared to the typical yields achieved previously. The cell weight acquired with the two protocols was similar, leading to the conclusion that it was the intracellular concentration of Hb that was increased with the improved protocol. Higher intracellular concentration will lead to more concentrated Hb solutions during all downstream steps, which is preferable to reduce dimer formation. Dimers of Hb are much more prone to autoxidize and deteriorate through subsequent globin destabilizing events (Zhang et al., 1991). Having higher recombinant Hb concentrations at the start of downstream purification processes will lead to higher protein quality in the end. The highest final purified yield in this work was achieved with the rHbFα4 mutant at 69 ± 8 mg Hb_[heme]_/L flask culture, which was just a little over 2-folds the yield of purified wt rHbF obtained in this study, 31 ± 7 mg Hb_[heme]_/L. We concluded that increasing negative net charge on the *α*-subunit appeared to significantly contribute to increased production of functional HbF in *E. coli* in this study. The yields of the rHbFγ2 and rHbFα4/γ2 mutants were lower, 16 ± 2 and 29 ± 12 Hb_[heme]_/L flask culture, respectively, due to the formation of non-functional HbF resulting in considerable losses. To confirm the benefits of the adapted flask culture protocol, we tried expressing three other HbF mutants reported elsewhere (Kettisen et al., 2021). Using the conventional purification protocol with cation exchange and anion exchange resins, we achieved yields of purified HbF (>95% purity) in the range ∼40–50 mg Hb_[heme]_/L flask culture (n > 3), in comparison to ∼15 mg/L reported previously (Ratanasopa et al., 2016). The rHbF double mutant αA12D/A19D with 50 ± 4 mg/L showed significantly improved yields compared to wt rHbF, which was not detected in the previous study (Kettisen et al., 2021). Nevertheless, these values are still low in terms of general recombinant protein yields, but for laboratory-scale Hb production, we demonstrate that even in flask cultures, unoptimized cultivation parameters lead to substantially more time and larger culture volumes needed to acquire sufficient amounts of Hb for characterization studies.

The fact that heme availability is imperative to yield and efficient expression of Hb has been established previously (Weickert et al., 1999) and thus it is not surprising to see an increased yield when the heme precursor ALA concentration was increased in the flask cultures. One aspect that is a bit more surprising is that higher temperatures led to higher yields as well. Previous studies have advocated the need for inducing the expression of Hb subunits at lower temperatures, due to instability of the expressed peptides at a physiological temperature (Weickert et al., 1997; Natarajan et al., 2011). However, we found that better yields were achieved at 35–37°C, as long as shaking speed and heme supply were sufficient to support the production. The increase in temperature might contribute to enhancing the cellular uptake of the heme precursor and thus allow for higher Hb concentrations.

Downstream purification of HbF mutants highlights the importance of sufficient separation power for detecting and removing non-functional Hb fractions from recombinant Hb. Purification of the negatively charged HbF mutants was successfully achieved by employing a multimodal ion exchange resin followed by the previously established anion exchange polishing step. The increase in the negative net surface charge of the HbF mutants examined in this work restricted the use of cation exchange chromatography conventionally used as the first step of Hb extraction from *E. coli* crude extracts (Reeder et al., 2008; Kettisen et al., 2018). Many soluble *E. coli* proteins present in the crude extract will not bind to a negatively charged resin at the proper chromatographic conditions, as the host cell proteome is skewed towards a negative net charge (Mu et al., 2017; Wennerström et al., 2020). This usually makes the cation exchange resin suitable for capturing a protein like Hb (pI ∼7) while washing away the bulk of host cell contaminating proteins. However, to adapt to the more negatively charged HbF variants produced in this work, we found a multimodal resin, which can function as a viable alternative for the more negatively charged mutants. In the range of conditions examined in this work, we also discovered that this resin had the useful trait of being capable of separating different fractions of Hb in the same crude extract. This is especially important when producing human Hbs.

With the ability of *ß*-type (β, γ) globins to form soluble tetramers, the tandem expression of the separate native Hb subunits always comes with the risk of forming a substantial portion of these non-functional complexes in recombinant HbA and HbF production (Ratanasopa et al., 2016). Unless properly separated, the presence of non-functional tetramers will affect the functionality and characterization of the sample. Ion exchange chromatography is a potent tool to optimize separation between isoforms of Hb proteins, as the subunits have diverse pI values, and we want to stress the importance of optimizing purification protocols and not overloading chromatographic columns during downstream procedures due to loss of resolution needed for separation.

As expected, we found that more mutations correlated with higher NaCl concentrations needed for elution from the Q HP anion exchange resin, supporting the conclusion that the selected mutations were surface-exposed as hypothesized and contributed to stronger interactions with the positively charged resin. The mutational strategy of switching/adding net surface charges on Hb was easily screened in this manner. The IEF determination correlated with the increased NaCl concentrations for elution, with lower pI values found in the corresponding order.

An aspect to keep in mind would be that the purification method with TREN was based on the rHbFα4/γ2 mutant, and it may be suggested that the protocol should be adjusted to better suit the other mutants in future studies. In terms of separation ability, the Q HP column may need additional optimization to achieve even better separation. In the end, elution conditions must be optimized according to the difference in pI of different Hb fractions within the same sample during ion-exchange chromatography and can be employed either on TREN, or Q HP. Regardless of the separation method used, the correct and target heterotetramer fraction of HbF can quickly be verified with ferricyanide and used for further characterization.

Autoxidation studies revealed a less oxidatively stable behavior of γ-subunit mutants. The autoxidation study showed that the two mutants harboring γ-subunit mutations had increased rates of spontaneous oxidation, while no significant difference was seen between rHbFα4 and wt rHbF. The spectral examination did not indicate any apparent structural perturbation to the heme pocket, as ligands could be bound and released without obvious adverse events, and oxidation in excess K_3_ [Fe(CN)_6_] proceeded without forming hemichrome species. The modified γ-subunit harbored the γK61E and γK82D mutations, and the latter γK82D was chosen due to previous reports indicating increased yield with the corresponding mutation in a HbA variant βK82D (Weickert et al., 1999). However, our results regarding autoxidation correlate with a report showing that the βK82D mutation increases the autoxidation rate (Strader et al., 2017). We have not included any additional oxidative studies in this work, but in future studies, it could be of interest to further examine the oxidative behavior of γK82D mutants to determine if this substitution in HbF would demonstrate protective traits seen in other important oxidative reactions. For example, reducing irreversible oxidation of hotspot βCys93 and increase resistance to oxidative degradation (Strader et al., 2017). One could also consider examining the EF7 site in γ for adding negative charge instead of EF6, as this site would correspond to the same location on the *α*-subunit as the αN78D mutation present in the rHbFα4 mutant, and examine if the autoxidation rate is less affected. The corresponding mutation would replace a glycine, and there have been reports of such a naturally occurring mutant in the *ß*-subunit: β83 (EF7) Gly→Asp, Hb Pyrgos (Tatsis et al., 1976).

We included only a few characterization experiments of the protein properties in this study. In further work, the impact of the negatively charged surface of these mutants will be examined in more detail. When designing mutants of Hb, any mutation causing increased oxidative reaction rates should be considered undesirable, as such reactions may cause several different toxic side-effects *in vivo* (Alayash 2017). For instance, oxidation of Hb can be associated both with increased heme losses (Kassa et al., 2016) and detrimental cellular inflammatory responses (Bozza and Jeney 2020). No adverse effect on the autoxidation rate was observed with the rHbFα4 mutant, which in addition showed significantly higher yields during production. Taken together, the rHbFα4 mutant should be considered as the most promising candidate out of the three HbF mutants in this study for further evaluations to elucidate its potential as a possible component in hemoglobin-based oxygen therapeutics.

## Conclusion

Three HbF mutants with an increased negative net charge at physiological pH were successfully produced in *E. coli*. Retention behavior shifts observed during chromatographic procedures strongly indicated the increased negative net surface charge on the HbF mutants. We determined the lower pI values for the mutants in comparison to wt by IEF experiments. The purification of the mutants was not trivial, and an alternative purification resin based on a multimodal modality was developed to be effective for isolating the variants, as well as to allow separation of non-functional Hb fractions. These non-functional Hb fractions were suggested to be made up of just one subunit, most probably only γ-subunits, by the means of denaturing IEF. This finding highlights the need for adequate purification strategies to separate non-target protein fractions, which should be of interest during any recombinant production of multi-subunit proteins where diverse subunit assemblies are possible. For the rHbFα4 where the mutated *α*-subunit was combined with the wt γ-subunit, a homogenous functional fraction of mutant HbF was achieved as opposed to the rHbFγ2 and rHbFα4/γ2 mutants. Thus, a more negatively charged *α*-subunit improved formation of the correct tetramer assembly, and production yields were enhanced accordingly. This mutant generated a flask culture yield of ∼70 mg Hb/L, which to our knowledge is the highest reported value for non-fused HbF production in shake flasks. Additionally, aside from the pI, the rHbFα4 mutant did not differ from wt rHbF in its basic biophysical properties, most importantly in terms of autoxidation. The rHbFγ2 and rHbFα4/γ2 mutants did, however, exhibit increased autoxidation rates, indicating that one or both of the γK61E and γK82D mutations may play a role in destabilizing the resistance to oxidation in HbF.

In summary, the strategy of increasing the negative net surface charge of HbF increases yields in recombinant production. However, unbalanced expression of the Hb subunits leads to loss of the target Hb fraction, and thus it may be more fruitful to initially use this strategy for improving the solubility of the *α*-subunit which is known to be less stable than *ß*-globins. The choice of mutation sites needs to be carefully considered to not disturb basic functionality and avoid causing increased rates of oxidative reactions. In this study, we based the selection mostly on previously reported naturally occurring mutants in HbVar, but through rational selection through other means, the surface of HbF could be modified for even more pronounced effects.

## Data Availability

The raw data supporting the conclusions of this article will be made available by the authors, without undue reservation.
